# Thoracoscopy for Spontaneous Pneumothorax

**DOI:** 10.3390/jcm10173835

**Published:** 2021-08-26

**Authors:** José M. Porcel, Pyng Lee

**Affiliations:** 1 Pleural Medicine Unit, Department of Internal Medicine, Hospital Universitari Arnau de Vilanova, 25198 Lleida, Spain; 2 Institut de Recerca Biomèdica de Lleida Fundació Dr. Pifarré, IRBLleida, 25198 Lleida, Spain; 3 School of Medicine, Universitat de Lleida, 25008 Lleida, Spain; 4 Division of Respiratory and Critical Care Medicine, The National University Hospital, Singapore 119228, Singapore; pyng_lee@nuhs.edu.sg

**Keywords:** thoracoscopy, VATS, spontaneous pneumothorax, bullectomy, pleurodesis

## Abstract

Video-assisted thoracic surgery (VATS) is the treatment of choice for recurrence prevention in patients with spontaneous pneumothorax (SP). Although the optimal surgical technique is uncertain, bullous resection using staplers in combination with mechanical pleurodesis, chemical pleurodesis and/or staple line coverage is usually undertaken. Currently, patient satisfaction, postoperative pain and other perioperative parameters have significantly improved with advancements in thoracoscopic technology, which include uniportal, needlescopic and nonintubated VATS variants. Ipsilateral recurrences after VATS occur in less than 5% of patients, in which case a redo-VATS is a feasible therapeutical option. Randomized controlled trials are urgently needed to shed light on the best definitive management of SP.

## 1. Introduction

Pneumothorax can occur spontaneously or because of trauma or procedural complication. Spontaneous pneumothoraces (SP) are divided into primary (PSP) and secondary (SSP). PSP occurs in someone without a known underlying lung disease, whereas SPP appears as a complication of an underlying lung disease, such as chronic obstructive pulmonary disease, lung cancer, interstitial lung disease, or tuberculosis. This distinction is probably artificial since most PSP patients have small subpleural emphysematous blebs and bullae (usually located in the lung apices) that may rupture, causing air to enter the pleural space, while others have an unrecognized lung disease (e.g., thoracic endometriosis, Birt–Hogg–Dubé syndrome, lymphangioleiomyomatosis, Langerhans cell histiocytosis, Ehlers–Danlos syndrome) [1]. Management of SP is guided by clinical symptoms, pneumothorax size and side (e.g., bilaterality), cause (PSP or SSP), occupation, and risk of recurrence. A recent meta-analysis of 29 studies comprising more than 13,500 adult patients with a first episode of PSP found that approximately 30% experienced recurrence within the first year, and females were at higher risk than males [2]. Of 170,929 hospital admissions in England for SP, 60.8% of patients had chronic lung disease [3]. The recurrence rate was 25.5% at 5 years, but it was higher in SSP than PSP (32% vs. 21%). Once a patient has a recurrence, subsequent recurrences are even more common. After a brief overview of the general management of SP and thoracoscopic techniques, this narrative review focuses on the role of thoracoscopy for the first and subsequent SP episodes.

## 2. Overview on the Management of Spontaneous Pneumothorax

Initial therapeutic options for PSP include observation with or without supplemental oxygen, manual aspiration with needle (14–16 G) or catheter (8–9 Fr), and chest catheters (≤14 Fr) or tubes (16–24 Fr) connected to either a water seal or an ambulatory drainage device [4,5,6]. Individuals with an SSP are primarily treated with catheter or tube thoracostomy (Table 1). Chest catheters (≤14 Fr) are recommended over chest tubes (>14 Fr) for both PSP and SPP, though some patients with SPP (e.g., hemopneumothorax, tension pneumothorax, barotrauma from mechanical ventilation, large air leaks) may benefit from large-bore chest tubes (24–28 Fr).

After the initial management of SP, the need for a definitive procedure to prevent recurrences should be evaluated (Table 2). In short, when a definitive procedure is indicated, video-assisted thoracic surgery (VATS) with stapling of blebs/bulla and pleurodesis is the treatment of choice [4,5,6]. Interventions should target the apical half of the thorax. In non-surgical candidates, chemical pleurodesis (e.g., talc, doxycycline) via chest tube represents an acceptable alternative. Ideally, the timing of these procedures should be within the same hospital admission as the risk of recurrence is highest during the first months [2].

### Should Thoracoscopic Surgery Be Offered for Every First Episode of PSP?

Some experts have suggested that thoracoscopy should be done at the first episode of PSP, irrespective of the circumstances highlighted in Table 2. The rationale is to reduce the patient’s anxiety and the economic healthcare burden related to a second episode. A meta-analysis of nine studies (1121 patients), of which only two were randomized controlled trials (RCT) (222 patients), showed that patients with a first episode of PSP have a more significant reduction in the ipsilateral recurrence rate when treated with VATS (irrespective of the type of surgical technique) than when treated conservatively (odd ratio 0.13) [7]. Specifically, for every three patients that undergo VATS operations, one recurrence is avoided (number needed to treat of 3.1 patients). One RCT recommended preventive VATS, particularly in those patients whose high-resolution computed tomography demonstrated bullae ≥ 2 cm [8]. However, the quality of current evidence on this debatable topic is moderate at best. On the other hand, if all patients were operated on after the first PSP occurrence, about two-thirds of them would undergo an unnecessary intervention; not to mention the potential, though few, side effects of surgery (e.g., postoperative bleeding, chest pain or paresthesia), and the increased technical difficulties for future thoracic surgeries. Ultimately, the preferred approach for an initial episode of PSP that does not meet the conditions outlined in Table 2 will be a decision shared with an adequately informed patient.

## 3. Thoracoscopic Techniques

**Figure 1:**
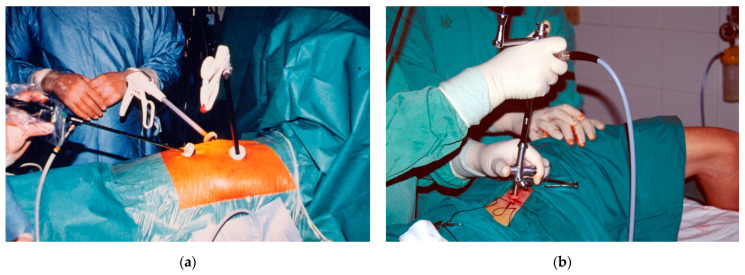
Thoracoscopic techniques. (**a**) Triportal VATS under general anesthesia; (**b**) Medical thoracoscopy under conscious sedation, using a rigid instrument.

Thoracoscopy, a procedure which allows access to the pleural space for diagnostic and therapeutic purposes, has classically been divided into “medical” and “surgical” [9,10]. Medical thoracoscopy (MT) is also referred to as pleuroscopy or local anesthetic thoracoscopy. It is usually performed by interventional pulmonologists in a non-operating room setting (e.g., endoscopy suite), under local anesthesia and moderate sedation. MT may be delivered via rigid or semi-rigid (flexi-rigid) instruments. Conversely, surgical thoracoscopy or VATS is conducted by a surgeon in an operating room, under general anesthesia with single lung ventilation and, traditionally, using three entry ports and rigid instruments (Figure 1). However, owing to technical advances, the boundary between medical and surgical thoracoscopy are becoming increasingly blurred. For example, nonintubated (spontaneous ventilation) uniportal VATS can be considered a “medical” variation of the original surgical procedure, which only has a few technical differences from classical MT. Table 3 provides a comparison of MT and VATS.

## 4. VATS for Spontaneous Pneumothorax

**Figure 2:**
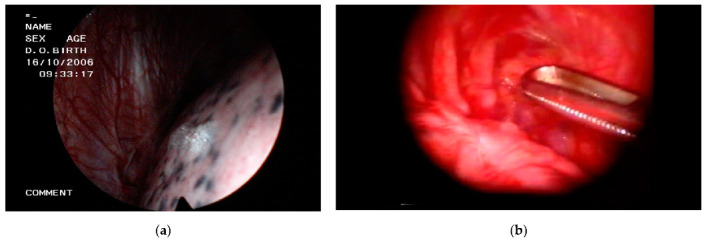
Thoracoscopic views in spontaneous pneumothorax. (**a**) Apical bleb; (**b**) Bleblectomy using an endostapler.

VATS (preferably) and thoracotomy are the two surgical approaches for the operative treatment of SP. During surgery, emphysema-like changes can be assessed in accordance with the Varderschueren classification (stage I, normal pleura; stage II, pleural adhesions; stage 3, blebs < 2 cm; and stage 4, bullae > 2 cm) [11]. If blebs and bullae are visible, which occurs in approximately 80% of the cases [5], they are generally resected (Figure 2) and then a pleurodesis procedure is undertaken. Even if macroscopic blebs/bullae are not apparent and no air leak is identified by a water or saline test, many surgeons proceed with lung apex excision, confident that emphysema-like changes will be discovered in the resected tissue [4,12]. However, under this specific situation (no endoscopic abnormalities and no air leak), others just prefer to apply talc poudrage [13].

Bullectomy/bleblectomy (also referred to as wedge resection) is mostly accomplished using an endostapler, but other alternatives, such as bulla suturing (no-knife stapler), endoloop ligation, and electrocoagulation (diathermy), exist. Pleurodesis can be achieved through different methods, whether mechanical (dry gauze abrasion above the fifth rib, apical or partial parietal pleurectomy, pleural electrocauterization), chemical (insufflation of talc or instillation of other chemical agent), mixed (staple line coverage of the dissected visceral pleura with an absorbable mesh [e.g., cellulose, Vicryl, polyglycolic acid] and/or fibrin glue), or a combination thereof.

It should be noted that there is great variability in the surgical treatment of SP among institutions and a lack of high-quality RCT to guide evidence-based management. A meta-analysis of 51 studies (only 2 RCT) comprising 6907 patients compared outcomes of different thoracoscopic interventions for PSP [14]. It was found that recurrence rates were lowest in the wedge resection plus chemical pleurodesis group (1.7%) and highest in the wedge resection alone group (9.7%), thus emphasizing the importance of combining interventions.

### 4.1. VATS or Medical Thoracoscopy?

Whilst the surgical management of SP is typically reserved for VATS, it might rarely be performed by skilled proceduralists using MT. For instance, in one study, 124 patients with PSP underwent electrocoagulation of blebs/bullae and talc poudrage pleurodesis under MT [15]. The mean operative time was about 15 min and only 4 (3%) patients required reoperation by axillary thoracotomy during follow-up. However, since complex parenchymal interventions, such as bullectomy/bleblectomy, are more appropriately undertaken at VATS, in clinical practice MT is reserved for cases where talc poudrage is simply selected as the method to prevent SP recurrences.

### 4.2. VATS or Open Thoracotomy?

VATS has gradually supplanted open thoracotomy and mini-thoracotomy and is now considered the standard definitive treatment of SP. VATS is minimally invasive and several meta-analyses have demonstrated that it results in a shorter operation time, less intraoperative blood loss, shorter hospital stays, fewer post-operative analgesic requirements, and better cosmesis than open surgery [16,17]. However, the risk of SP recurrence following VATS is higher when compared to thoracotomy, which justifies the use of supplemental procedures during surgery (i.e., pleurodesis) as previously stated. The higher postoperative recurrence rate with VATS can be attributed to a higher chance of missed leaking blebs and a less intense pleural inflammatory reaction induced by this technique than by thoracotomy. Overall, the frequency of SP recurrence following VATS are reported to range from about 4% to 11%, whereas it is approximately 1% with open thoracotomy [18,19].

A French national database comprising 7396 SP patients, of whom 977 (13%) were treated by open thoracotomy and 6419 (87%) by a three-port VATS technique, offers comparative data between both procedures [20]. Although the proportion of PSP and SSP was unreported, roughly 40% of patients had underlying respiratory conditions that could predispose to SSP. Surgical procedures consisted of bullectomy (57% in open surgery and 66% in VATS) and pleurodesis (100%), the latter being performed mainly by mechanical abrasion or apical pleurectomy (79%) or, less commonly, by using a chemical agent (21%). There was a significantly higher recurrence rate of SP after VATS (3.8% vs. 1.8%), with a median time to recurrence of 3 months. Hospital length of stay was reduced by an average of one day in patients subjected to VATS, while the frequency of pulmonary complications also favored this technique (8% vs. 12%) [20].

A recent national-level epidemiologic study in the United States included 21,838 SSP admissions during 2016 and 2017 [21]. Despite guideline recommendations, only 7366 (33.7%) received prophylaxis of SSP recurrence during the same hospitalization, largely by VATS (80.8%). The 90-day post-discharge recurrence rates were similar for VATS and open surgery (4.10% and 4.03%, respectively). However, the chance of developing a recurrent SSP was four to five times higher in patients who received medical pleurodesis alone.

### 4.3. Uniportal or Multiportal VATS?

VATS is classically performed using two or three ports. Experience with single-incision VATS is increasing, though still limited. A meta-analysis of 17 retrospective case-control studies examined 502 SP patients who underwent uniportal VATS and 486 treated with a three-port VATS procedure [22]. The uniportal variant, as compared with the three-port VATS, did not increase mortality, recurrence rates (4.34% vs. 4.79%), operative time (61 vs. 59 min) or postoperative hospital stay (5.71 vs. 5.84 days), but significantly reduced patient postoperative pain and paresthesia, and improved patient satisfaction. The largest series from a single center on the use of uniportal VATS for SP included 351 patients [23]. The authors proved the feasibility and safety of this technique, which had a recurrence rate of 3.6% and resulted in 85% patient satisfaction due to the single small scar. To our knowledge, there is only one RCT in which 135 PSP patients were recruited and treated by either a single, double, or three-port approach (45 in each branch) [24]. The study indicated that uniportal VATS was less painful and had better cosmetic results, while it yielded similar efficiency as the two or three-port variants (overall recurrence rate 5%). Despite these advantages, single-access VATS is not yet widely used for SP. One reason may be that it requires greater technical skill to manage surgical instruments within small confines. In fact, a study suggested at least 100 procedural experiences for proficiency [25].

### 4.4. VATS or Needlescopic VATS?

For surgeons with insufficient experience to perform a uniportal VATS, but who would like to offer patients the benefits of this surgical modality, needlescopic VATS emerges as a reasonable alternative. For example, in a retrospective comparison of 106 PSP patients who underwent needlescopic VATS and 89 who were managed with conventional VATS, the former procedure was significantly associated (like uniportal VATS) with less postoperative pain and minimal skin scarring (3 mm wounds) [26]. Needlescopic VATS has never been compared with classical VATS in a RCT.

### 4.5. Intubated or Nonintubated VATS?

VATS generally involves endotracheal intubation under general anesthesia, which is inevitably associated with a risk of complications related to major airway injury (e.g., sore throat, hoarseness, tracheal damage) and the residual effects of muscle relaxants. It is feasible, however, to perform SP surgery using intravenous and/or locoregional anesthesia in a spontaneously breathing patient; the so-called nonintubated or awake VATS. Nonintubated VATS is a suitable alternative for patients who cannot receive general anesthesia because of increased risks [27]. Only three RCT that respectively enrolled 43, 41, and 335 patients have evaluated the safety and feasibility of this procedural adaptation [28,29,30]. The largest one assigned half the patients to nonintubated VATS and the other half to mechanical ventilation and found that awake VATS hastened the recovery from surgery, decreasing the operative consumption of intravenous opioid analgesia and the overall cost of anesthesia [30]. The other two small RCT highlighted the shorter operative and perioperative time in the awake group [28,29].

### 4.6. VATS Pleurodesis

Several techniques can be used to induce pleural symphysis in SP patients subjected to VATS, with significant variations among surgeons, hospitals, and countries. Notably, the normal parietal pleural surface in SP patients tends to be excruciatingly painful during a pleurodesis procedure, thus making adequate pain control necessary in the days or weeks after the intervention.

#### 4.6.1. Mechanical or Chemical Pleurodesis?

A meta-analysis of one RCT and 6 observational cohort studies tried to ellucidate the best pleurodesis method, whether mechanical or chemical, following bullectomy for PSP [31]. Of 1933 PSP patients (mean age of around 27.5 years), 1032 were treated with mechanical pleurodesis and 901 with chemical pleurodesis. Mechanical pleurodesis consisted of pleural abrasion (*n* = 799), pleurectomy (*n* = 202) or both (*n* = 31), whereas chemical pleurodesis was performed predominantly with talc (*n* = 643) or, less commonly, minocycline (*n* = 69) or others. Chemical pleurodesis was superior in reducing recurrence rates (1.2% vs. 4%) and hospital stay (by 0.42 days). The reason for this superiority may be a matter of a more extensive distribution of the chemical agent throughout the pleural surface. Another meta-analysis of 5 studies (3 RCT and 2 retrospective) aimed to determine which VATS pleurodesis approach, single intervention (mechanical) or a combined intervention (mechanical and chemical), is more effective in preventing SP recurrence [32]. The combined group included 561 patients and the mechanical group 286. Adding a chemical agent to mechanical pleurodesis provided a 63% lower risk of devoloping a recurrent SP compared to single intervention, though at the expense of an increasing rate of postoperative pain.

Finally, although a previous pleurodesis for SP neither makes patients unsuitable for lung transplantation nor significantly affects surgical outcomes [33,34], some experts prefer mechanical over chemical pleurodesis in transplant candidates, in the belief that talc may be associated with difficulties during surgical reintervention.

#### 4.6.2. Mechanical Pleurodesis: Abrasion or Pleurectomy?

Partial pleurectomy entails a parietal pleura stripping, whereas abrasion involves the rubbing of the parietal pleura, using a gauze or brush, until petechial bleeding occurs. A meta-analysis of 6 RCT examined pleural abrasion and other procedures in PSP patients [35]. As compared with apical pleurectomy, pleural abrasion had advantages in terms of operative time, postoperative bleeding, and residual chest pain and discomfort, although both equally reduced the recurrence of PSP. In addition, abrasion is simpler and less technically demanding than pleurectomy.

#### 4.6.3. Chemical Pleurodesis: Talc

Talc is the most commonly used sclerosing agent worldwide. Tetracycline derivatives (e.g., minocycline, doxycycline) are alternative options in countries where talc is unavailable. In a large prospective series of 1415 patients with PSP undergoing VATS, with intervention to bullae in half the cases and talc poudrage (3–4 g) in all, the incidence of recurrent SP was only 1.9% after a median follow-up period of 8.5 years [13]. Of note, bullae suturing or ligation (instead of resection) were associated with a significantly higher frequency of recurrence (3.8% and 15%, respectively) compared with subjects receiving talc poudrage alone (0.3%). A subsequent systematic review of 8 studies (*n* = 2324), of which only one was randomized, confirmed that PSP recurrence following VATS with talc poudrage was very low (0% to 3.2%) [36]. The same article reviewed 4 additional studies (*n* = 249) in which talc poudrage was insufflated through MT without intervention on the lung, which provided higher recurrence rates of between 2.5% and 10.2% [36].

For patients unable or unwilling to undergo VATS or MT, pleurodesis via chest catheter using talc slurry (or other sclerosing agent) is a possibility. However, talc slurry is less effective than talc poudrage for SP because of the ability of the latter to target the lung apex where most SP originate.

### 4.7. Staple Line Coverage

Covering the staple line area after bullectomy/bleblectomy reinforces the visceral pleura and also has a symphyseal effect. One prospective study randomized 1414 PSP patients who underwent bullectomy with staplers to a coverage group (*n* = 757) in which the staple line was covered with an absorbable cellulose mesh and fibrin glue, or to a mechanical abrasion group (*n* = 657) [37]. Both groups showed comparable recurrence rates at 1 year (9.5% and 10.7%, respectively), but patients in the mechanical group had significantly more residual pain. In another RCT of 204 PSP patients who required VATS bullectomy and apical pleural abrasion, half were assigned to receive Vicryl mesh to cover the staple line and the other half were not (control group) [38]. There was a reduction in postoperative SP recurrences at 1 year in the mesh group (2.9% vs. 15.7%). Finally, according to a meta-analysis of 8 studies (3 RCT and 5 retrospective), totalling 1095 SP patients who were subjected to bullectomy, staple line coverage with a bioabsorbable polyglycolic acid patch also resulted in a lower postoperative recurrence rate (3.7% vs. 15.3%) [39].

### 4.8. Redo-VATS

Experience is scarce on the optimal approach to recurrent SP following VATS or thoracotomy [40,41,42,43]. In a Korean series of 188 patients in whom PSP recurred after VATS, 76 (40%) underwent redo VATS surgery, 60 (32%) were treated by observation, and 52 (28%) by tube thoracostomy [42]. A subsequent recurrence was seen in 3%, 20%, and 33% of the treatment groups, respectively, but these figures were 2.9%, 68%, and 57% in a smaller series of 34 patients [41]. This emphasizes that redo-VATS is probably the best option in this particular population, unless pneumothorax size is minimal. The typical operative findings at redo-VATS are pleural adhesions (70–80%) and the presence of blebs/bullae (90%) which predominate on the staple line or are new and have a different location from those seen at the original VATS [41,42]. Minor postoperative complications develop in about 10% of the cases. In patients who previously received talc pleurodesis, pleural adhesions may be more dense and, therefore, redo-VATS may be more challenging.

## 5. Conclusions

The indication for a definitive procedure to prevent recurrences of SP should be based on the probability of new episodes, patient profession and preferences, and procedural aspects (e.g., risks, surgeon’s skills). VATS is the preferred operative approach. Combining stapled bullectomy/bleblectomy with a single (e.g., abrasion, partial pleurectomy, talc poudrage, staple line coverage) or double pleurodesis method results in very low recurrence rates. However, there is no consensus on the best treatment of blebs and bullae, except when they are leaking, nor on the ideal method of pleurodesis. In patients who are inoperable or refuse surgery, bedside pleurodesis via chest catheter/tube is recommended. Recurrences following VATS can be managed with redo-VATS.

## Figures and Tables

**Table 1:** Initial treatment of spontaneous pneumothorax.

Clinical Scenario		Management
A.	First episode of PSP, no dyspnea, and small size on chest radiograph ^1^	Observation with supplemental oxygen
B.	First episode of PSP, dyspnea, or large size on chest radiograph	Needle aspiration or chest catheter/tube
C.	First episode of PSP, and severe dyspnea or hemodynamic instability regardless of size	Immediate drainage with chest catheter/tube. Emergent needle decompression if tension physiology
D.	First episode of bilateral PSP	Chest catheter/tube
E.	First episode of PSP, with no resolution after observation or needle aspiration	Chest catheter/tube
F.	First episode of PSP, associated with a pleural effusion	Chest catheter/tube
G.	Recurrent PSP	Chest catheter/tube
H.	SSP	Chest catheter/tube

^1^ ≤3 cm at apex or ≤2 cm at hilum. PSP, primary spontaneous pneumothorax; SSP, secondary spontaneous pneumothorax.

**Table 2:** Indications for definitive management and prevention of recurrences in spontaneous pneumothorax.

Type of Spontaneous Pneumothorax	Indications for Definitive Therapy
PSP, first episode	Tension pneumothorax Persistent air leak >5–7 days High risk professions or hobbies ^1^ Bilateral pneumothorax Patient desire for definitive therapy Concomitant indication for thoracoscopy ^2^
PSP, second episode	All cases
SPP	All cases ^3^

^1^ Airline pilots, divers. Sailors can also be included because they will not have immediate medical access if an SP episode develops at sea. ^2^ Hemopneumothorax, lung biopsy. ^3^ Exceptions include patients who refuse treatment, or a first episode of a small asymptomatic SPP. PSP, primary spontaneous pneumothorax; SSP, secondary spontaneous pneumothorax.

**Table 3:** Medical thoracoscopy versus surgical thoracoscopy.

Feature	Medical Thoracoscopy	VATS
Proceduralist	Interventional pulmonologist	Thoracic surgeon
Location	Endoscopy suite or operating room	Operating room
Anesthesia	Local, conscious sedation ^1^	General, single lung ventilation, double-lumen endotracheal tube
Entry ports	One	Two or three
Instruments	Rigid ^2^, flex-rigid	Rigid
Technical variants	Mini-thoracoscopy ^3^, flex-rigid thoracoscopy ^4^	Uniportal ^5^, needlescopic ^6^, nonintubated
Indications in SP patients	Pleurodesis, electrocoagulation of blebs	Bullectomy/blebectomy, pleurodesis, staple line coverage

^1^ Midazolam or dexmedetomidine in combination with fentanyl usually provide good sedation and analgesia. ^2^ The diameter of the rigid thoracoscope most commonly used is 7–10 mm. ^3^ Telescope of 3.3–5.5 mm. ^4^ Similar in handling to flexible bronchoscope, the pleuroscope has a proximal rigid and a flexible distal part, and 7 mm in outer diameter. ^5^ The thoracoscope and other instruments (e.g., stapler, grasper) are introduced through a single 2–2.5 cm skin incision. ^6^ Needlescopic VATS uses the existing chest drain wound as a working port and adds two 3-mm ports. SP, spontaneous pneumothorax; VATS, video-assisted thoracic surgery.
